# Paracetamol Use in Patients With Osteoarthritis and Lower Back Pain: Infodemiology Study and Observational Analysis of Electronic Medical Record Data

**DOI:** 10.2196/37790

**Published:** 2022-10-27

**Authors:** Gisèle Pickering, Linda Mezouar, Hayet Kechemir, Caty Ebel-Bitoun

**Affiliations:** 1 Centre d'Investigation Clinique Inserm 1405 CHU Clermont-Ferrand Clermont-Ferrand France; 2 Sanofi Specialty Care Gentilly France; 3 Sanofi Research and Development Chilly-Mazarin France; 4 Sanofi Consumer Healthcare Gentilly France

**Keywords:** osteoarthritis, lower back pain, general practice, rheumatology, paracetamol, real-world evidence

## Abstract

**Background:**

Lower back pain (LBP) and osteoarthritis (OA) are common musculoskeletal disorders and account for around 17.0% of years lived with disability worldwide; however, there is a lack of real-world data on these conditions. Paracetamol brands are frequently prescribed in France for musculoskeletal pain and include Doliprane, Dafalgan, and Ixprim (tramadol-paracetamol).

**Objective:**

The objective of this retrospective study was to understand the journey of patients with LBP or OA when treated with paracetamol.

**Methods:**

Three studies were undertaken. Two studies analyzed electronic medical records from general practitioners (GPs) and rheumatologists of patients with OA or LBP, who had received at least one paracetamol prescription between 2013 and 2018 in France. Data were extracted, anonymized, and stratified by gender, age, and provider specialty. The third study, an infodemiology study, analyzed associations between terms used on public medical forums and Twitter in France and the United States for OA only.

**Results:**

In the first 2 studies, among patients with LBP (98,998), most (n=92,068, 93.0%) saw a GP, and Doliprane was a first-line therapy for 87.0% (n=86,128) of patients (71.0% [n=61,151] in combination with nonsteroidal anti-inflammatory drugs [NSAIDs] or opioids). Among patients with OA (99,997), most (n=84,997, 85.0%) saw a GP, and Doliprane was a first-line therapy for 83.0% (n=82,998) of patients (62.0% [n=51,459] in combination). Overall, paracetamol monotherapy prescriptions decreased as episodes increased. In the third study, in line with available literature, the data confirmed that the prevalence of OA increases with age (91.5% [212,875/232,650] above 41 years), OA is more predominant in females (46,530/232,650, 20.0%), and paracetamol use varies between GPs and rheumatologists.

**Conclusions:**

This health surveillance analysis provides a better understanding of the journey for patients with LBP or OA. These data confirmed that although paracetamol remains the most common first-line analgesic for patients with LBP and OA, usage varies among patients and health care specialists, and there are concerns over efficacy.

## Introduction

### Background

Musculoskeletal disorders are associated with chronic and debilitating pain, account for 17% of all years lived with disability worldwide, and are particularly prevalent in higher income countries (Western Europe, 20.8%; United States, 25.8%; Japan, 26.2%) [1]. The recurrent pain experienced can have considerable consequences, causing anxiety and generally negatively impacting a patient’s quality of life [2]. With the global population of individuals over 60 years of age expected to increase significantly by 2050 [3], management of these disorders, which often progress with age, is particularly important. Two of the most common conditions causing musculoskeletal pain are lower back pain (LBP) and osteoarthritis (OA) [4].

### LBP

LBP, the most common musculoskeletal disorder, is experienced by approximately 38.0% of the general population, and is the leading cause of disability worldwide [5,6]. A previous study has shown that most patients experience mild LBP (25.0%), with severe LBP experienced by the least patients (5.0%) [7]. With rates increasing in the United States by 62.3% between 1992 and 2006, and more patients seeking assistance from health care professionals (HCPs), the prevalence appears to be increasing [8]. LBP can result from acute trauma, such as strenuous physical activities like lifting, pushing, or pulling a heavy load, with pain intensity ranging from sharp shooting pain, arising during sudden strenuous activity, to chronic pain that develops slowly over time as a result of degenerative changes within the spinal cord [9,10]. However, the vast majority of cases are classified as acute nonspecific, as no definitive cause can be identified, and this will be the focus of this study [11]. Frequently, nonspecific LBP is acute or subacute in nature and may resolve within days to a few weeks; however, LBP is considered chronic if the pain persists for more than 12 weeks [12].

For patients with acute nonspecific LBP, effective treatment remains a challenge [12,13]. Management is often patient centered and involves patient education and reassurance. Nonpharmacological options, such as physical exercise and manual techniques, are usually considered prior to pharmacological therapies. For chronic LBP, multidisciplinary care, involving a number of specialists, may also be considered. Paracetamol or nonsteroidal anti-inflammatory drugs (NSAIDs) are usually the first-line pharmacological options. If ineffective, stronger medications, such as opioids, antidepressants, and gabapentinoids, can be used; however, the risk of an adverse reaction is higher, and regular patient re-evaluation is required. Invasive surgery is usually the last option and is considered on a case-by-case basis (Table 1) [12,14-19].

**Table 1:** Guideline recommendations for acute lower back pain.

Guideline	Disease management options in the guideline
High Authority of Health Recommendation of Good Practice Management of a patient with common low back pain [19]	First-line option: Patient information and self-management Second-line option: Physical activity, paracetamol, and nonsteroidal anti-inflammatory drugs Third-line option: Multidisciplinary approach Fourth-line option: Biopsychosocial
French Society of Rheumatology (Société Française de Rhumatologie) How is low back pain or common low back pain treated? [18]	First-line option: Reassurance Second-line option: Pharmacological and nonpharmacological pain treatment Third-line option: Physical activity and psychosocial therapy
National Institute for Health and Care Excellence (NICE) Guideline Low back pain and sciatica in over 16s: assessment and management [17]	First-line option: Patient information and physical activity Second-line option: Nonsteroidal anti-inflammatory drugs and paracetamol in combination with weak opioids Third-line option: Specialist referral
Noninvasive treatment for acute, subacute, and chronic low back pain: A clinical practice guideline from the American College of Physicians (ACP) [12]	First-line option: Nondrug therapies such as physical activity, acupuncture, relaxation, and biofeedback Second-line option: Nonsteroidal anti-inflammatory drugs Third-line option: Tramadol or duloxetine Fourth-line option: Opioids

### OA

OA is characterized by the progressive destruction of articular cartilage associated with subchondral bone remodeling, formation of osteophytes, and secondary inflammation of synovial membranes [20,21]. Pain, mediated by a number of factors, including innervation and vascularization of the articular cartilage, is a principal symptom of OA [21,22]. Compressive forces, along with hypoxia, are believed to stimulate the development of nerves, causing pain even after inflammation has subsided [23]. OA-related pain includes both nociceptive and nonnociceptive components [24], and is associated with abnormally excitable pain pathways in the peripheral and central nervous systems [21].

Symptomatic knee OA is one of the more common forms of OA, experienced by approximately 22.9% of individuals over 40 years, and is a significant cause of disability [25,26]. In France, current OA management guidelines recognize the need for a combinational approach, using both pharmacological and nonpharmacological treatments tailored to each patient. First-line pharmacological options include paracetamol and NSAIDs, followed by opioids, symptomatic slow-acting drugs, and duloxetine (off-label use). Topical agents and intra-articular treatments have also been recommended (Table 2) [27]. Furthermore, these patients often have comorbidities, which may increase the risk of drug-drug interactions and limit the range of drugs that can be used. Age-associated differences in drug sensitivities should also be considered when treating older patients [28,29].

**Table 2:** Guideline recommendations for osteoarthritis.

Guideline	Disease management options in the guideline
French Society of Rheumatology (Société Française de Rhumatologie) Recommendations on the pharmacological treatment of knee osteoarthritis [27]	First-line option: Nonsteroidal anti-inflammatory drugs (paracetamol if nonsteroidal anti-inflammatory drugs are contraindicated) Second-line option: Weak/strong opioids Third-line option: Symptomatic slow-acting drugs Fourth-line option: Duloxetine
National Institute for Health and Care Excellence (NICE) Osteoarthritis: care and management [30]	First-line option: Patient information, self-management, and thermotherapy Second-line option: Exercise, manual therapy, weight loss, and electrotherapy Third-line option: Pharmaceutical management (eg, creams, paracetamol, nonsteroidal anti-inflammatory drugs, and opioids) Fourth-line option: Surgery
American College of Rheumatology/Arthritis Foundation Guideline for the management of osteoarthritis of the hand, hip, and knee [31]	First-line option: Topical or oral nonsteroidal anti-inflammatory drugs alongside physical, psychosocial, and mind-body approaches Second-line option: Intra-articular glucocorticoid injections, paracetamol, and duloxetine Third-line option: Tramadol

### The Role of Paracetamol

Given the similarities in the initial treatment recommendations for both LBP and OA, paracetamol is the most commonly employed first-line analgesic for both conditions, used by over 94.4% of patients with LBP and over 96.0% of patients with OA [32-35]. There are a variety of paracetamol brands available in France, and Doliprane is the most prescribed paracetamol (Table 3) [36,37]. This is backed up by an infodemiology study analyzing 44,283 social media posts, in which Doliprane was the most mentioned paracetamol with 31.7% of posts, followed by Dafalgan with 10.9% of posts and Ixprim with 9.8% of posts [38].

The use of long-term analgesia for patients over 65 years remains challenging, and HCPs must weigh the benefits and risks given the potential increased risk of adverse effects in these patients [39,40]. A high proportion of patients discontinue prescription analgesics within the first few months due to inadequate pain relief or intolerable side effects [41,42]. With few clinical trials enrolling patients over 65 years and fewer incorporating diverse races and ethnicities, there is limited data available for patients over 65 years. Infodemiology, a relatively new method allowing real-word data collection, may help fill the knowledge gap [28]. In this project, infodemiology encompasses electronic medical records (EMRs), interactive online medical forums, and social media (Twitter), allowing analysis of this publicly available, previously unexplored, valuable source of data [43].

As the prevalence of these diseases is expected to increase with the aging population, having a thorough understanding of the requirements of patients who seek treatment is important and may assist in the development of gold-standard care. This retrospective project was designed to provide an insight into the journey of French patients with OA or LBP treated with paracetamol.

**Table 3:** Chemical and brand names of drugs evaluated for use in lower back pain and osteoarthritis.

Chemical name	Brand name
Betamethasone	Diprostene
Diclofenac	Unbranded Voltarene
Diclofenac epolamine	Flector
Gabapentin	Neurotonin
Ketoprofen	Bi-Profenid
Paracetamol	Dafalgan Doliprane Unbranded
Paracetamol-codeine	Klipal
Paracetamol- dextropropoxyphene	D-Antalvic
Paracetamol-opium	Lamaline
Tramadol-paracetamol	Ixprim

## Methods

### Project Design

Two retrospective noninterventional studies were performed in France, using EMRs, with a focus on patients with LBP and OA. These studies were distinct, and the data were analyzed separately, but the methods used to generate the results were the same.

The third study, an OA open-source study, used social listening. For this, publicly available data were analyzed, focusing on patients with OA, from both the United States and France.

For all 3 studies, the data collected were used to identify usage of analgesics in anonymized patients with either LBP or OA. This retrospective project analyzed data extracted from EMRs provided by doctors working in the community and discussions on social media. As such, no efficacy or safety data, or reasons for termination of medication were collected.

### Population Characteristics and Patient Journey Data Sources

For the EMR studies, prescription data for approximately 3 million people were obtained from general practitioners (GPs) and rheumatologists in France. Comprehensive data on diagnoses, prescriptions, referrals, physician visits, and laboratory results allowed for analysis of pharmacological and nonpharmacological therapies. For patients with OA, data were collected between September 2013 and August 2018, and the following criteria were applied: (1) the patient must have visited a GP or rheumatologist between September 2013 and August 2018; (2) the patient must have at least one OA diagnosis, defined by International Classification of Diseases Version 10 (ICD-10) codes M15-M19; and (3) the patient must have at least one paracetamol prescription. For patients with subacute, acute, or chronic LBP, data were collected between October 2013 and September 2018, and the following criteria were applied: (1) the patient must have visited a GP or rheumatologist between October 2013 and September 2018; (2) the patient must have at least one LBP diagnosis, defined by ICD-10 code M54.5; and (3) the patient must have at least one paracetamol prescription. Distinguishing between different brands of paracetamol was not possible, so drugs were separated by brand name in order to understand usage patterns and distinguish preference.

Details on drugs prescribed in addition to paracetamol were extracted from the database. The drugs included in the analysis were the most frequently used and approved drugs for the treatment of either OA or LBP. For OA, this included ketoprofen, opioids, and NSAIDs in combination with opioids and some intra-articular steroidal injections. For LBP, this included ketoprofen, opioids, and NSAIDs in combination with opioids, intra-articular steroidal injections, antiepileptic drugs, and some antidepressants. In some cases, details of dose and the type of concomitant medication were not collected and therefore not reported in this analysis.

### OA Open-Source Study

The OA open-source study, which focused on both French and United States populations, gathered data from only publicly available sources. Questions, comments, and posts related to back pain and OA from both English and French language public medical forums and Twitter were extracted into a text data set. For the United States only, data were extracted for the year 2016 from The Centers for Disease Control and Prevention National Health Interview Survey (CDC NHIS), which comprehensively captures major aspects of a respondent’s health and wellness condition; however, only relevant data, such as demographics, occupation, income bracket, and major medical conditions, were included.

### Endpoints

The endpoint of this project was to define the demographics of patients with OA and LBP, and develop an understanding of the management of these conditions in France, including how prescribing methods differ between GPs and rheumatologists. In addition, this project aimed to evaluate the use of paracetamol in patients with OA and LBP within France.

### Data Management and Transformation

For the EMR studies, anonymized patient data provided by the Decision Resources Group (DRG) [44] were loaded into the querying platform (Snowflake platform version 3.12.0), and analysis was performed using Structured Query Language. The following transformations and operations were applied to the data: demographics; comorbidities and clinical profiling; treatment persistence; and patient journey.

Patients were stratified by gender and age, as well as provider specialties. Comorbidities and clinical profiling were assessed by interrogating the medical history data that triggered an entry related to a prescription, procedure, or diagnosis in the physician’s office. Medical history data were captured as part of a patient’s anamnesis. Diagnostic data were captured on an ongoing basis, and medical history data and current diagnoses were consolidated to map a patient’s comorbidity profile.

To take into account gender and age, medical history and diagnostic data were assessed to determine the frequency and distribution of diagnoses based on ICD-10 codes (either for OA [M15-M19] or LBP [M54.5]), and these data were then further segmented into gender and age groups (10-year increments).

Treatment persistence was assessed for patients who had received a paracetamol prescription. For this, the first paracetamol prescription was defined as the initiation of therapy, and longitudinal prescription data were then assessed to account for further paracetamol prescriptions over time. For patients with more than 60 days without a prescription, defined by the difference between the first prescription plus days of supply and the subsequent prescriptions, 2 separate pain therapies were reported. Prescription for another main medication, either a different brand or active compound, was considered termination of the preceding pain medication and considered a switch event; however, no distinction between concomitant and new agents was made. Treatment duration was assessed and visualized across the entire population and analyzed in smaller cohorts based on age, gender, and comorbidity profile.

Assessment of the patient journey involved analyzing pain medication dynamics for patients with either OA or LBP across the entire patient population, as well as subcohorts over time. Switching dynamics assessed the share for each pain medication brand by month. This also segmented the patient share for “new” patients, who had no preceding pain medication prescription over the observation period; “switch” patients, who received other pain medication brands and whose treatment was switched during the respective month; and “repeat” patients, who were prescribed the same brand as in the preceding month. Where the volume of data was sufficient for product brands, the patient share and uptake rate analyses were segmented across age, gender, and comorbidity subcohorts.

For the OA open-source study, Python’s Natural Language Toolkit library was employed to extract nonrelevant discussions and responses from public medical forums and Twitter data, leaving only the key relevant “talking points,” which were then broken down into key words and phrases for analysis. For the CDC NHIS data, all unanswered fields in a record were floored to zero, categorized features were label encoded and dummied to separate columns, and prevalence of OA was selected as the target variable.

### Statistical Methods

All analyses conducted in this work were descriptive, and no other confirmatory statistical tests were applied. For the EMR studies, patient data were primarily summarized with percentages, which were calculated using Excel (Microsoft Corp). In some instances, the median for the duration in days was calculated. The data are reflective of what was presented within the EMRs, and no missing values were imputed. Internal DRG therapeutic area experts who specialized in OA and LBP reviewed the results of the analysis for the respective OA or LBP data. For this analysis, in some cases, absolute numbers were recalculated from percentages.

For the OA open-source study, graph/network analysis was applied to study pairwise relationships between the following: symptom-condition, condition-treatment, symptom-medicine, symptom-treatment, symptom-diet and supplements, and symptom-activities. The relationship between 2 terms was determined by their co-occurrence in the same comment or post.

### Ethical Considerations

This noninterventional work was performed on fully deidentified and anonymized patient EMRs, and the methodology did not require any human interaction. Therefore, no notifications to the National Competent Authorities or Ethics Committee were required by national and local regulations and requirements.

## Results

### LBP

#### LBP Population Characteristics

In the first EMR study, 98,998 patients with LBP were included. The majority (n=69,299, 70.0%) of the patients were between 21 and 60 years, and 54.0% (n=53,459) were female (Table 4). Most (92,068/98,998, 93.0%) patients were treated by a GP, with acute and subacute LBP being the most common complaints. Many patients presented with comorbidities, including additional musculoskeletal system disorders, and respiratory system and digestive system disorders.

**Figure 1:**
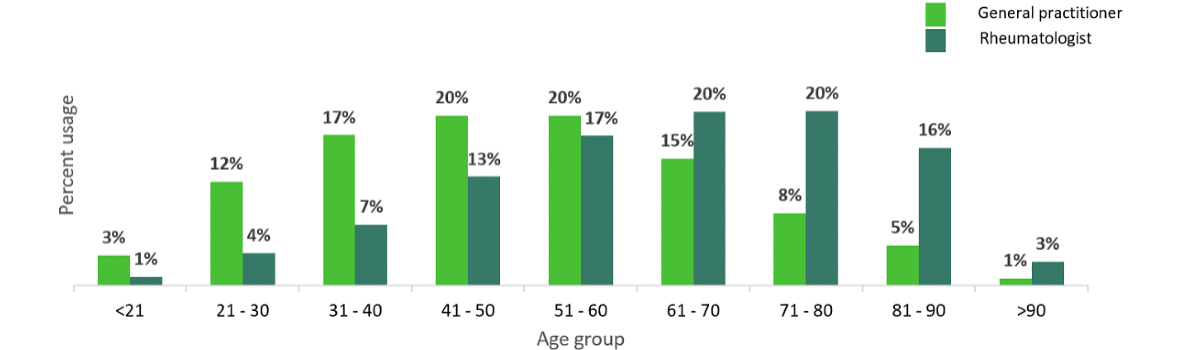
Distribution of patients with lower back pain by age group and specialist setting.

The distribution by age and specialty showed that the difference in distribution was highest in patients aged 21-40 years compared with those aged 61-90 years, who had a higher percentage of visits to the rheumatologist (Figure 1).

**Table 4:** Patient characteristics for the electronic medical record–based studies.

Baseline demographics		Lower back pain^a^ (N=98,998)	Osteoarthritis^a^ (N=99,997)
**Age (years), n (%)**			
	<40	30,689 (31.0)	1000 (1.0)
	41-50	18,810 (19.0)	3000 (3.0)
	51-60	18,810 (19.0)	12,000 (12.0)
	61-70	14,850 (15.0)	24,999 (25.0)
	71-80	8910 (9.0)	27,999 (28.0)
	81-90	4950 (5.0)	23,999 (24.0)
	>90	990 (1.0)	6000 (6.0)
**Gender, n (%)**			
	Female	53,459 (54.0)	67,998 (68.0)
	Male	45,539 (46.0)	31,999 (32.0)
**Care setting, n (%)**			
	General practitioner	92,068 (93.0)	84,997 (85.0)
	Rheumatologist	6930 (7.0)	15,000 (15.0)

^a^Absolute numbers were recalculated from percentages.

#### Patient Therapeutic Journey

Paracetamol prescription was required for inclusion into the study. Among those included, 87.0% (86,128/98,998) received the Doliprane paracetamol brand as first-line therapy and 71.0% (61,151/86,128) were taking it in combination with other drugs, such as NSAIDs or opioids.

Of the 12.0% (10,335/86,128) of patients who switched from the Doliprane brand to a second-line therapy, 52.0% (5374/10,335) received an alternative paracetamol product, of which, Lamaline, Dafalgan, and Ixprim were equally prescribed (all n=215, 4.0%). Voltarene (397/4961, 8.0%) was the most popular option for the 48.0% (4961/10,335) of patients who switched to a nonparacetamol product as second-line therapy, followed by Bi-Profenid (198/4961, 4.0%).

Of the 13.0% (12,870/98,998) of patients who received a non-Doliprane brand paracetamol as first-line therapy, 57.0% (7336/12,870) switched to Doliprane as second-line therapy, and 43.0% (5534/12,870) of those taking a nonparacetamol product as first-line therapy switched to Doliprane as second-line therapy. Of those taking Doliprane as second-line therapy, 69.0% (5062/7336) were using it in combination with other drugs, such as NSAIDs or opioids.

**Figure 2:**
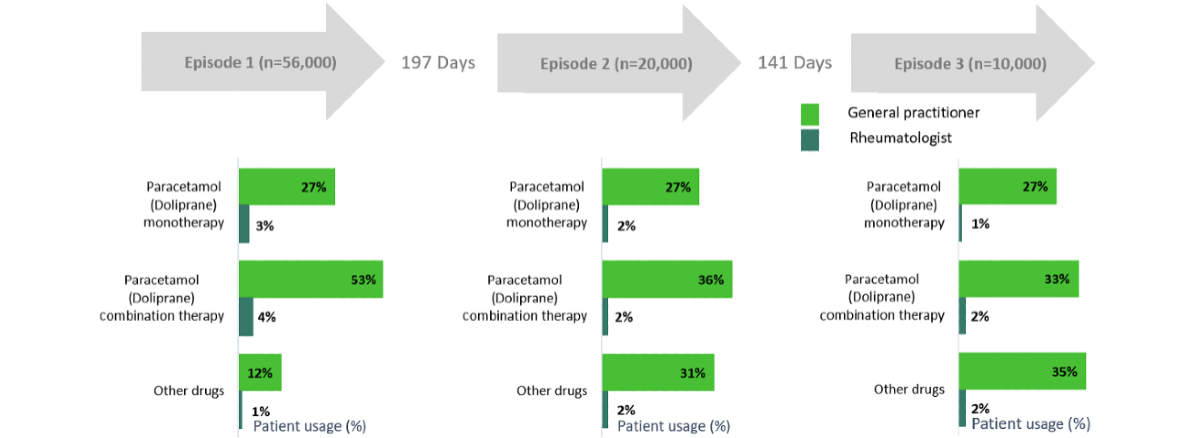
Distribution of patients with lower back pain on Doliprane versus other medications by episode.

Assessing the choice of treatment by episode and setting, the prescribing patterns of paracetamol as a monotherapy by GPs appeared consistent over multiple episodes; however, their use of paracetamol combination treatments decreased over time and the use of other drugs increased (Figure 2).

Reviewing the top 5 brand paracetamol agents used for the treatment of LBP, Doliprane was the most used (approximately 57.0% [52,440/92,000] and 64.0% [3840/6000] for GPs and rheumatologists, respectively), with GPs making more use of unbranded paracetamol and Bi-Profenid, compared with rheumatologists, who favored Flector and Diprostene (Table 5). For rheumatologist prescriptions, the use of Doliprane as monotherapy and combination therapy decreased over time.

Overall, 90.0% (89,098/98,998) of all paracetamol products prescribed were branded and 79.0% (57,670/73,000) of all nonparacetamol medications were branded.

**Table 5:** The top 5 agents prescribed for patients with lower back pain by general practitioners and rheumatologists.

Drug used	General practitioner^a^ (N=92,000), n (%)	Rheumatologist^a^ (N=6,000), n (%)
Paracetamol (Doliprane)	52,440 (57.0)	3840 (64.0)
Paracetamol (Dafalgan)	26,680 (29.0)	1620 (27.0)
Paracetamol (Voltarene)	16,560 (18.0)	480 (8.0)
Paracetamol (unbranded)	12,880 (14.0)	N/A^b^
Ketoprofen (Bi-Profenid)	11,040 (12.0)	N/A
Diclofenac epolamine (Flector)	N/A	600 (10.0)
Betamethasone (Diprostene)	N/A	540 (9.0)

^a^Absolute numbers were recalculated from percentages.

^b^N/A: not applicable.

### OA

#### OA Population Characteristics

In the second EMR study, 99,997 patients with OA were included, of which, 96.0% (n=95,997) were over 50 years, with most between 71 and 80 years (n=27,999, 28.0%), and 68.0% (n=67,998) were female (Table 4). The majority (84,997/99,997, 85.0%) of cases were seen by a GP, of which, approximately 60.0% (50,998/84,997) were for diffuse OA or unspecified OA; in comparison, 67.0% (10,050/15,000) of cases seen by a rheumatologist were for knee OA.

#### Patient Therapeutic Journey

Doliprane as a prescribed paracetamol brand was the most common analgesic of those analyzed among both GP and rheumatology specialties, being the first-line therapy for 83.0% (82,998/99,997) of patients. Among patients who received Doliprane as a prescribed paracetamol first-line therapy, 62.0% (51,459/82,998) used it in combination with another drug. Of the 13.0% (10,790/82,998) of patients who used Doliprane as a prescribed paracetamol first-line therapy and switched to an alternative therapy, 30.0% (3237/10,790) moved to an alternative paracetamol product, with the most common being Dafalgan (194/3237, 6.0%), followed by Lamaline (129/3237, 4.0%), while 70.0% (7553/10,790) moved to a nonparacetamol product, with the most common being diclofenac (Voltarene) (906/7553, 12.0%), followed by Flector (453/7553, 6.0%). Among the patients who switched from a non-Doliprane drug (n=16,999) as a prescribed first-line therapy, 30.0% (5100/16,999) of those taking paracetamol moved to Doliprane as second-line therapy, and 70.0% (11,899/16,999) of those taking a nonparacetamol product moved to Doliprane as second-line therapy. Among those for whom Doliprane was a prescribed paracetamol brand as a second-line therapy, 63.0% (10,709/16,999) were using it as a combination therapy.

**Figure 3:**
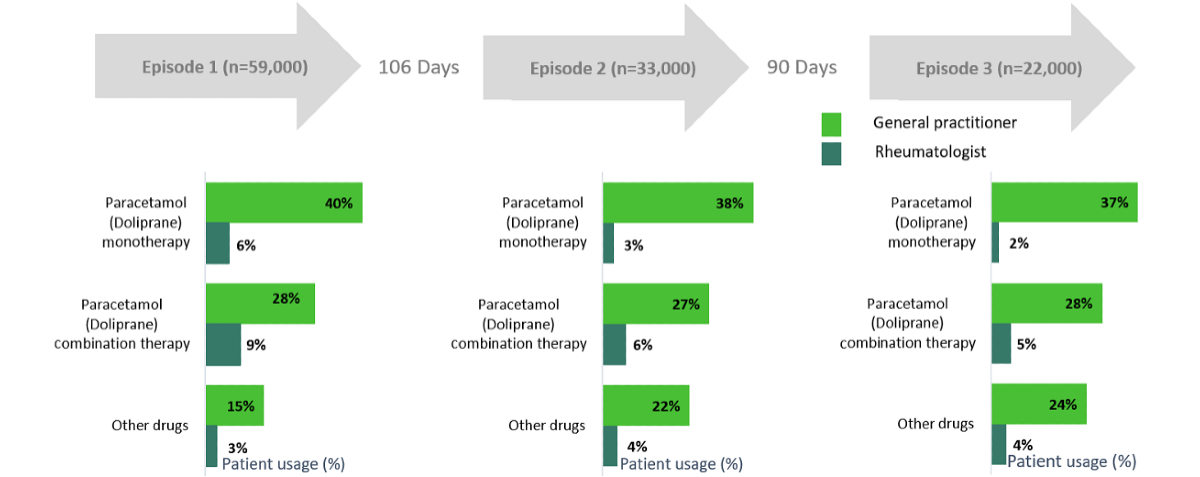
Distribution of patients with osteoarthritis on Doliprane versus other medications by episode.

GPs showed fairly consistent prescribing patterns, with Doliprane as monotherapy being the most common choice across episodes; however, rheumatologist prescribing patterns were comparatively less consistent (Figure 3).

**Figure 4:**
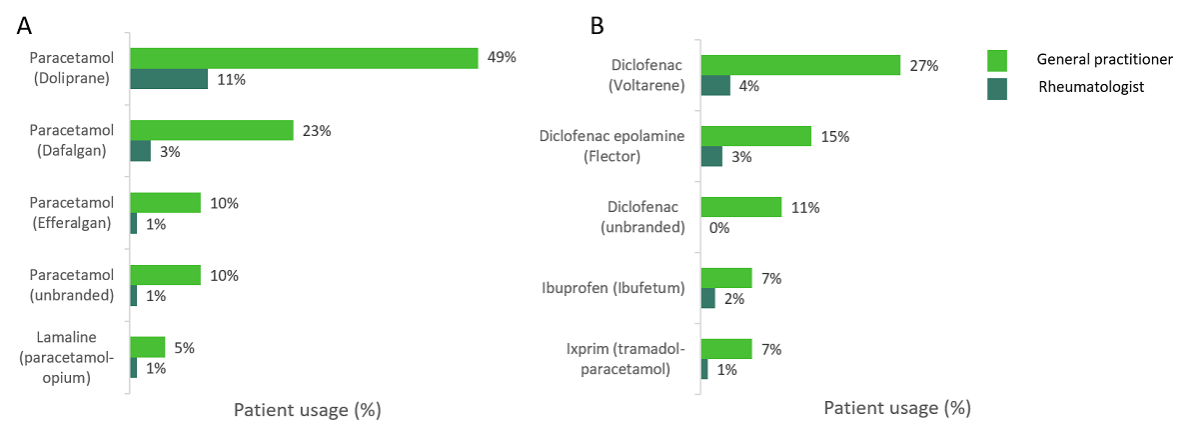
The top 5 agents among (A) paracetamol and (B) nonparacetamol treatments for patients with osteoarthritis.

Reviewing the top 5 paracetamol agents prescribed overall for OA, Doliprane as a prescribed brand was the most popular in both GP (48,999/99,997, 49.0%) and rheumatologist (11,000/99,997, 11.0%) settings, followed by Dafalgan (Figure 4). Regarding nonparacetamol agents, Voltarene was prescribed in 27.0% (15,120/56,000) of patients by GPs and 4.0% (2,240/56,000) by rheumatologists, followed by Flector (Figure 4). Of the paracetamol products prescribed, 92.0% (91,997/99,997) were branded, and of the nonparacetamol products prescribed, 86.0% (48,160/56,000) were branded. Overall, paracetamol was favored over NSAIDs for both LBP and OA; however, NSAID use was higher in patients with OA.

### OA Open-Source Study

#### Population Characteristics

The OA open-source study, using data obtained by social listening, showed that OA occurrence increases with age, that is, approximately 91.5% (212,875/232,650) of patients were over 41 years. These data suggest that the overall incidence of OA is 17.3% (40,248/232,650) and that OA is more predominant in females (46,530/232,650, 20.0%) than males (32,571/232,650, 14.0%). Furnishing workers, communications equipment operators, and workers in military-specific occupations reported the highest incidence of OA, whereas life and physical scientists and computer specialists reported the lowest rates. There is an indication that both smoking and alcohol consumption may influence the overall risk of OA. The most common comorbidities reported, including circulatory and respiratory system disorders, are shown in Multimedia Appendix 1.

#### Patient Therapeutic Journey

According to the data obtained from medical forums, pain was the most commonly reported reason for using medication, with anti-inflammatory drugs and analgesics mentioned the most. Specific drugs, such as tramadol, Doliprane, Dafalgan, and Voltarene, were also mentioned. The discussion around pain medications shifted from analgesics and NSAIDs, to stronger medications, such as morphine, Lamaline, Di-Antalvic, Neurontin, and Klipal, as pain intensity increased.

**Figure 5:**
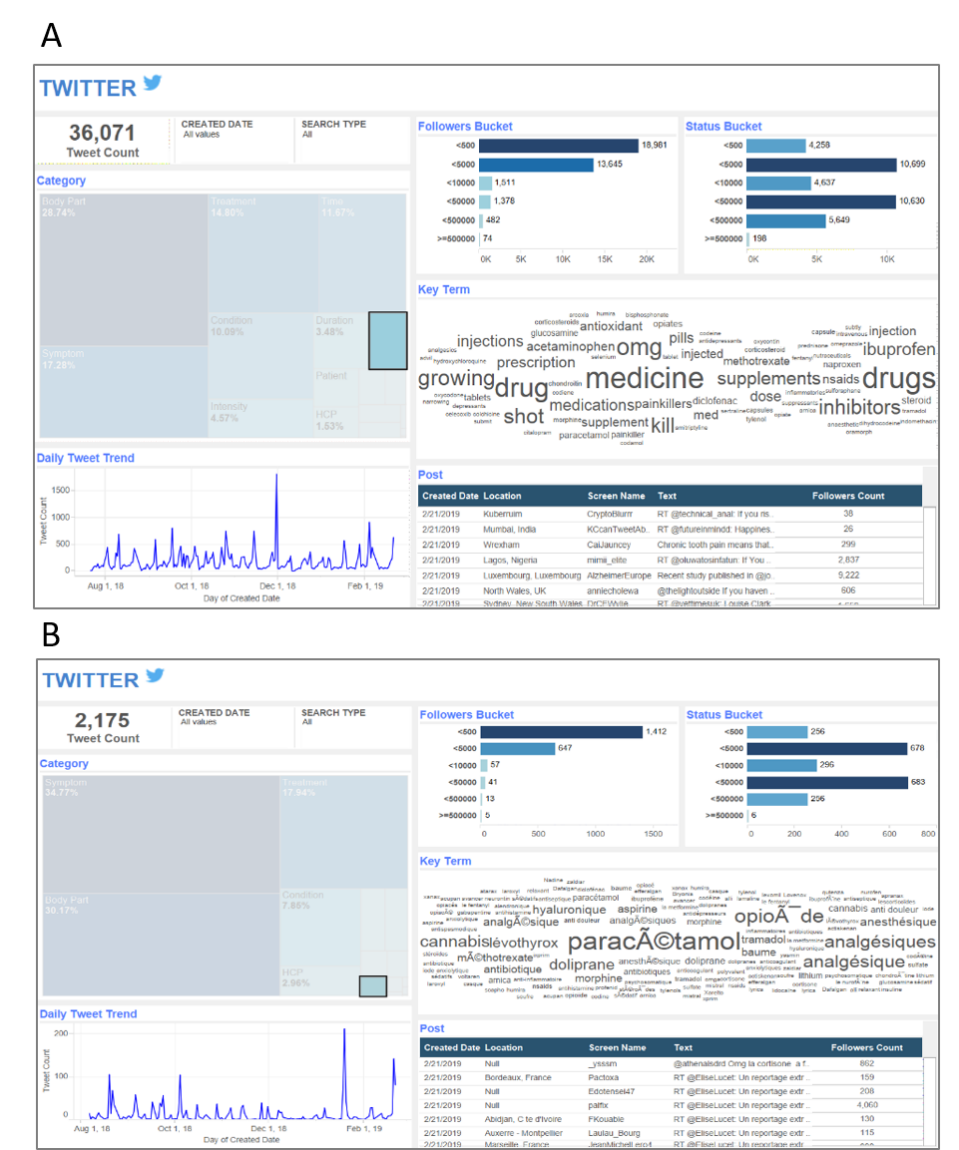
The most commonly used medications for osteoarthritis-related pain according to Twitter in (A) English (United States) and (B) French (France). Information was collected through the automated metrics provided by each of the social media monitored during the study.

From the 285,315 posts discussing when to seek medical advice on Twitter, the primary reasons identified were inflammation, pain, overweight, stiffness, and swelling. Evidence also suggested that noninvasive nonpharmacological options, such as massage therapy, are generally preferred by patients for the management of OA-related pain. Of the 36,071 posts discussing pain medications in the context of back pain and OA, 2175 posts revealed that paracetamol, analgesics, and opioids were the most frequently discussed (Figure 5). Furthermore, posts from France were more likely to mention specific drug names or classes, whereas posts from the United States contained general terms related to medicine.

## Discussion

This overview of analgesic usage in France analyzed data obtained from GP and rheumatologist practice settings, along with information extracted from publicly available sources, such as medical forums and Twitter, in France and the United States. The data presented within this project provides several insights into the journey of patients with LBP and OA.

In this project, across all 3 studies, the proportion of women experiencing LBP or OA was higher than that in men. In line with this finding, a study in France assessing chronic musculoskeletal pain found a higher prevalence in women (41%, 95% CI 40.2-42.4) than in men (29%, 95% CI 28.0-30.3) [45]. We showed that the prevalence of OA increases with age, with the majority of patients being over 60 years. This was expected, owing to the degenerative nature of this disease that progresses with age [46,47].

With 70.0% of the patients with LBP aged between 21 and 60 years, lifestyle and physical workload have been considered factors associated with an increased risk of LBP [48]. Data obtained from patient records indicated that a higher percentage of patients with LBP and OA visited GPs as opposed to rheumatologists, likely due to the need for a referral in order to see a specialist. Additionally, older patients (>61 years) were more likely to visit a rheumatologist rather than a GP, possibly due to pre-existing comorbidities (eg, arthritis).

The top 5 treatments prescribed by GPs and rheumatologists differed between conditions, with diclofenac use more prevalent in patients with OA, possibly reflecting the differences in practice guidelines. However, these variations could also be attributed to the different underlying pain mechanisms involved (eg, chronic vs acute pain), with chronic inflammation predominant in patients with OA [49].

In both EMR studies, the majority of prescriptions, both for paracetamol and nonparacetamol, were branded, possibly due to the majority of patients in France using national insurance to cover medical costs [50]. In both cohorts, increasing episodes correlated with increased drug use over time and a shorter duration between episodes, indicating that patients who need repeat visits require stronger medication to manage disease progression.

In the OA open-source study, social listening, a relatively new approach, was used to gain better insights into OA prevalence and the treatment strategies employed by patients with OA across France and the United States. Due to instant accessibility and ease of use, many patients turn to online interactive medical forums or other social media platforms to discuss medical issues and treatment strategies. Social listening allows the exploration of this potentially valuable source of information [51,52]. Analysis of thousands of medical forums suggested that OA occurs in approximately 17.3% of the general population in the United States. These data also indicated that the majority of patients with OA are over 41 years, and that the incidence is higher in females (20.0%) than males (14.0%). Pain appeared to be the main reason prompting patients to seek medical care and for the initiation of pharmacological treatment, with both anti-inflammatory drugs and analgesics commonly discussed. With increasing pain, the discussion shifted to stronger medications, such as morphine, Lamaline, Di-Antalvic, Neurontin, and Klipal; however, social media posts suggested that patients prefer nonpharmacological options, such as massage.

Notably, patients in France were more likely to tweet about specific drug classes (eg, NSAIDs and opioids) and drug names (paracetamol), whereas patients in the United States were more likely to tweet general terms, such as medicine and medication. Paracetamol was well recognized as a first-line therapy for both OA and LBP [53-55], and a 2005 international study that reviewed the opinions of HCPs found that 82.0% of rheumatologists and 90.0% of French GPs recommended paracetamol as first-line therapy for OA. In this project, 76.0% of patients interviewed were taking paracetamol for OA and a further 39.0% of patients had switched from an NSAID due to side effects [56].

On the other hand, contrary evidence on the efficacy of paracetamol for musculoskeletal disorders has been published. A randomized double-blind study by Williams et al [32] compared paracetamol use with placebo for the treatment of acute back pain in 1643 participants and found no effect on recovery time. The median time to recovery was 17 days (95% CI 14-19) for those who took paracetamol regularly, 17 days (95% CI 15-20) for those who took paracetamol as needed, and 16 days (95% CI 14-20) for the placebo group [32]. Additionally, a meta-analysis review that included 1785 cases reported that paracetamol did not produce better outcomes than placebo for patients with LBP, including for scores of sleep, quality of life, or physical function [57]. Interestingly, a 2005 meta-analysis review found that paracetamol use was an effective pain management approach for patients with chronic pain, with a favorable safety profile compared with NSAIDs, recommending that paracetamol be used as an NSAID sparer [58]. Another meta-analysis review of 10 randomized controlled trials, including 1712 patients with OA, found that while NSAIDs were more effective than paracetamol for pain relief, paracetamol was still effective [59]. Conversely, paracetamol in combination with weak opioids (eg, tramadol) has been proven to be effective in managing pain symptoms associated with OA [60]. In addition to being clinically ineffective in the treatment of LBP and OA, use of paracetamol can result in abnormal liver function test results compared with placebo [61].

The conflicting evidence on the efficacy of paracetamol may, in part, be due to the different types of pain experienced by patients. Nociplastic pain, defined as pain occurring through altered nociception without nociceptor activation and nerve injury, is characterized by widespread pain through multiple body regions and often causes ongoing pain symptoms [60]. However, neuropathic pain is caused by nerve damage and often does not respond to certain medications. As such, it is important to identify the type of pain, as different therapies may be needed for appropriate management [62]. Given the conflicting evidence around the benefits of paracetamol for musculoskeletal disorders, current guidelines for the treatment of OA and LBP now vary on whether paracetamol is recommended as a first-line therapy.

Multiple guidelines, including the 2016 recommendations from the National Institute of Clinical Excellence (NICE) and the 2017 American College of Physicians clinical guidelines, advise against the use of paracetamol alone as a first-line therapy for acute LBP [17,55,63-65]; however, paracetamol is still recommended as an effective treatment in some guidelines, including the 2019 American College of Rheumatology guidelines for the management of OA and the 2019 Osteoarthritis Research Society International (OARSI) guidelines for the nonsurgical treatment of knee OA [66]. Nevertheless, it is clear from the data in this project that many physicians are still prescribing paracetamol both alone and in combination with other drugs as first- and second-line therapy.

Pain, a significant symptom for both OA and LBP, is complex and multifaceted, and is influenced by many aspects of a patient’s body and environment. It is affected by, but not limited to, their mood, sleep patterns, and avoidance behavior [2,67,68]. The relationship between HCPs and the patients who experience musculoskeletal pain is important, with guidelines recommending that they engage in shared decision-making, taking into consideration the needs of the patients, as well as their comorbidities, preferences, and values [69], and ensuring that patients receive enough information related to their condition in a clear and concise manner [70]. Consequently, it is important to not only address pain medication needs, but also ensure that the patient’s specific needs are addressed throughout the patient journey. It is hoped that the data in this project may provide insights into the patient journey, to assist physicians by highlighting prescribing patterns, so that they may make informed decisions when treating their patients.

The limitations associated with obtaining data through EMRs include the fact that only information recorded by the HCP is available for analysis, providing a possible information bias, as the EMR may not always contain accurate information because it relies on the patients to provide factual reports of their condition and medication consumption to the physician. Additionally, the data were not adjusted to account for disease severity. Furthermore, while treatment switches were recorded, details on the reasons for treatment switches were not available due to a lack of physician notes. It should also be noted that different patient identification methods are used within the EMRs, depending on the patient setting, which may mean that only part of a patient’s journey is captured. Since these data did not record self-medication with over-the-counter drugs or the dose of paracetamol prescribed, the actual consumption of analgesics in these patients could be higher.

The use of EMRs within this project had advantages with the supply of data from multiple settings. The project period allowed review of the treatment journey over multiple years, and included recent data for visibility of current trends. The use of social listening also allowed access to extensive information, which could lead to better understanding of the real-world management of LBP and OA. Combining these methodologies provided a substantial overview of a patient’s journey, allowing for the analysis of treatment selection and providing a better understanding of the health care system and its strengths and weaknesses overall.

Overall, this exploratory study used infodemiology, a new data collection approach, to confirm the currently available literature on the epidemiology of LBP and OA, and investigate commonly used treatment strategies. In France, LBP and OA are prevalent musculoskeletal conditions, with OA predominantly affecting patients over 60 years of age and LBP predominantly affecting patients between 21 and 60 years of age; in both groups, the majority of patients are female. Although the use of paracetamol as a first-line analgesic to treat patients with OA and LBP is quite common, its efficacy is debatable. Usage variability between GPs and rheumatologists suggests that OA severity differs among settings. These data will provide clinicians, pharmacists, and patients with a better understanding of the usage of analgesic medications within these settings, and aid in the understanding of prescriber behavior in the real-world setting when treating musculoskeletal disorders.

## Appendix

Multimedia Appendix 1 Comorbidity conditions recorded for patients.

## Abbreviations

CDC: Centers for Disease Control and Prevention
DRG: Decision Resources Group
EMR: electronic medical record
GP: general practitioner
HCP: health care professional
ICD-10: International Classification of Diseases Version 10
LBP: lower back pain
NHIS: National Health Interview Survey
NSAID: nonsteroidal anti-inflammatory drug
OA: osteoarthritis
