# Supplementary material for: Paracetamol Use in Patients With Osteoarthritis and Lower Back Pain: Infodemiology Study and Observational Analysis of Electronic Medical Record Data
Source: JMIR Public Health Surveill. 2022 Oct 27;8(10):e37790. doi: 10.2196/37790 (PMC9650576; doi:10.2196/37790)
Supplement: Multimedia Appendix 1 [file publichealth_v8i10e37790_app1.docx]

Multimedia Appendix 1. Comorbidity conditions recorded for patients

| Comorbidity | Occurrence of OA among patients suffering from this condition n (%) |
| --- | --- |
| Obesity (BMI >30) | 58,163 (25.0) |
| Coronary heart disease | 95,387 (41.0) |
| Hypertension | 74,448 (32.0) |
| Asthma | 62,816 (27.0) |
| Chronic bronchitis | 100,040 (43.0) |
| COPD | 109,346 (47.0) |
| Liver failure | 97,713 (42.0) |
| Kidney failure | 107,019 (46.0) |

Abbreviations: BMI, body mass index; COPD, chronic obstructive pulmonary disease;
OA, osteoarthritis
